# An Improved SELEX-Seq Strategy for Characterizing DNA-Binding Specificity of Transcription Factor: NF-κB as an Example

**DOI:** 10.1371/journal.pone.0076109

**Published:** 2013-10-10

**Authors:** Guangming Gu, Tingting Wang, Yang Yang, Xinhui Xu, Jinke Wang

**Affiliations:** The State Key Laboratory of Bioelectronics, Southeast University, Nanjing, China; Université Paris-Diderot, France

## Abstract

SELEX-Seq is now the optimal high-throughput technique for characterizing DNA-binding specificities of transcription factors. In this study, we introduced an improved EMSA-based SELEX-Seq strategy with several advantages. The improvements of this strategy included: (1) using a FAM-labeled probe to track protein-DNA complex in polyacrylamide gel for rapidly recovering the protein-bound dsDNA without relying on traditional radioactive labeling or ethidium bromide staining; (2) monitoring the specificity of SELEX selection by detecting a positive and negative sequence doped into the input DNAs used in each round with PCR amplification; (3) using nested PCR to ensure the specificity of PCR amplification of the selected DNAs after each round; (4) using the nucleotides added at the 5′ end of the nested PCR primers as the split barcode to code DNAs from various rounds for multiplexing sequencing samples. The split barcode minimized selection times and thus greatly simplified the current SELEX-Seq procedure. The reliability of the strategy was demonstrated by performing a successful SELEX-Seq of a well-known transcription factor, NF-κB. Therefore, this study provided a useful SELEX-Seq strategy for characterizing DNA-binding specificities of transcription factors.

## Introduction

In the human genome, there are about 2000 transcription factors (TFs) [1]–[3]. In these TFs, approximately more than 700 are DNA-binding TFs [3], [4]. These DNA-binding TFs can bind to various DNA binding sites (DBSs) in genome and regulate the transcription of their target genes. To elucidate the complete function of these proteins, it is essential to identify their complete *in vivo* DBSs. However, the current techniques based on chromatin immunoprecipitation (ChIP), such as ChIP-Seq [5]–[7], are incapable to determine the definite DBSs of these TFs. Therefore, identification of *in vivo* DBSs with the support of DNA-binding specificity characterized *in vitro* has become more and more important [8]–[12]. In the past ten years, the protein-binding microarray (PBM) has been successfully used to characterize the *in vitro* DNA-binding specificities of many TFs [13]–[20], so that the DNA-binding specificities of about 406 TFs have already been characterized with this technique [21], [22]. However, this technique suffered from the high cost of PBM and the insufficient coverage of all possible sequences over the length of 12 nucleotides.

Systematic evolution of ligands by exponential enrichment (SELEX) is an evolutionary process that allows screening the ligands that can be specifically bound by a target of interest from an initially random pool of aptamers [23]–[26]. This technique has been extensively used to screen the DNA-binding sequences of many TFs [27]–[30]. However, the traditional SELEX technique depended on very low-throughput cloning DNA sequencing technology, only dozen of sequences were obtained by most of SELEX studies. Subsequently, the SELEX-SAGE technique was developed to improve the throughput so that it reached to hundreds or thousands of sequences [31]. In recent years, with the development of massive parallel DNA sequencing techniques, SELEX-Seq was developed as a high-throughput SELEX technique. In the past four years, this technique has already been successfully used to characterize the high-resolution DNA-binding specificity of dozens of TFs. For example, in 2009, Zykovich *et al.* determined the *in vitro* binding sites and relative affinities of TF Zif268 and Aart by using SELEX-Seq for the first time [32]. In 2010, Jolma *et al.* characterized the DNA-binding specificities of 19 TFs belonging to 14 different classes by using SELEX-Seq [33]. In 2011, Slattery *et al.* characterized the DNA-binding specificities of heterodimers formed by eight homoeobox (Hox) TFs with a cofactor extradenticle (Exd) by using SELEX-Seq, and found that Exd binding to the Hox proteins modulated the sequence specificity of these proteins [34]. More recently, Wong *et al*. characterized the DNA-binding specificities of four dimers of NF-κB family (p52p52, p65p50, p65p52 and p65p65), and found that NF-κB dimers could specifically bind non-canonical motifs [35]. These studies demonstrated that SELEX-Seq is a mighty technique for characterizing the DNA-binding specificities of TFs at present.

The procedure of SELEX-Seq experiment mainly consists of three steps: (i) selecting dsDNAs specifically bound by a TF protein from a random library by SELEX, (ii) sequencing the selected dsDNAs with massive parallel DNA sequencing technique (such as Illumina Solexa), and (iii) bioinformatics analysis of sequence data. In the reported SELEX-Seq studies, the strategies for implementing these steps were not identical, especially those for the first step. This step is critical for the success of SELEX-Seq study. The key to this step is how successfully selecting DNA targets specifically bound by TF protein of interest from the random library. In the traditional SELEX studies, the main strategies for this step were to employ electrophoresis mobility shift assay (EMSA) [29], [36], affinity chromatography [37], and filter-binding assay [38], [39]. In the reported SELEX-Seq studies, EMSA [32], [34], [35] and affinity chromatography [32], [33] were employed. In the affinity chromatography approach, the protein-bound DNA was isolated with a special solid support (such as maltose resin column [32] and streptavidin-coupled plate [33]) that was coupled with TF proteins fused special tags (such as MBP [32] and SBP [33]). In the EMSA approach, the protein-bound DNA was isolated from the free DNA by running the DNA/protein binding reaction with native polyacrylamide gels. In comparison, the EMSA approach needs no special chemically modified supports and tagged TF proteins. In addition, the formation of DNA/protein complex can be directly monitored in the EMSA approach. Therefore, the EMSA approach was employed by many traditional SELEX-cloning sequencing and the current SELEX-Seq studies [32], [34], [35]. Although the present SELEX-Seq procedures worked well, these procedures still suffered from some limitations that may damage their applicability. Therefore, the improvement of the current SELEX-Seq procedures is still in need.

In this work, we introduced an improved EMSA-based SELEX-Seq strategy for characterizing DNA-binding specificity of TFs in a more rapid, specific and cost-effective manner. This strategy was featured with the following functions: (i) recovering the protein-bound DNA from the native polyacrylamide gels with a support of FAM-labeled probe; (ii) monitoring protein binding specificity by PCR detection of a positive and negative sequence added into the input DNA; (iii) ensuring the specific PCR amplification of the selected DNA by using the nested primers; and (iv) reducing selections by using split barcode added at the 5′ end of PCR primers. The reliability of the strategy was validated by performing a successful SELEX-Seq of a well-known transcription factor, NF-κB.

## Results

### Schematic Presentation of SELEX-Seq Strategy

**Figure 1:**
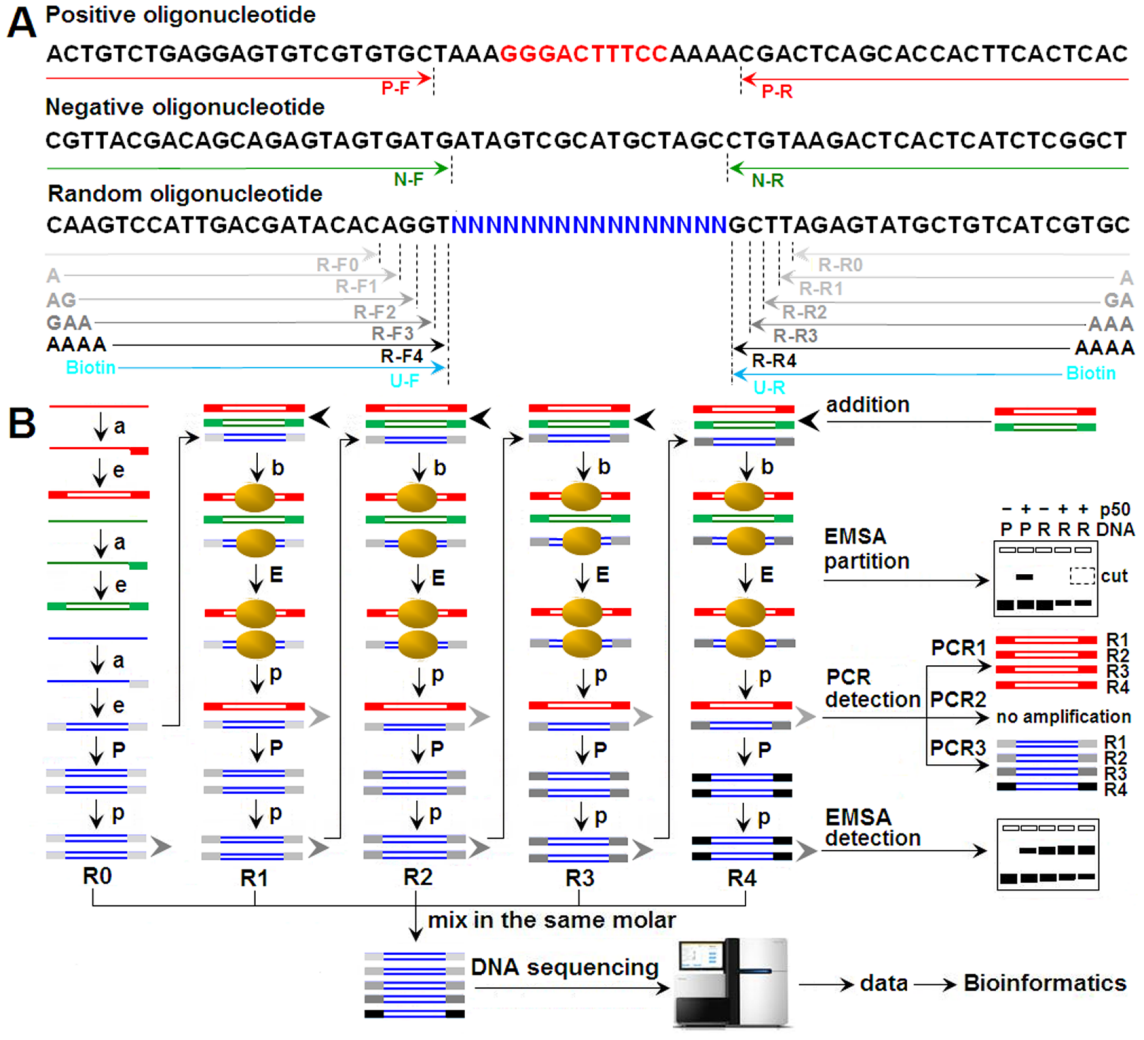
Schematic presentation of SELEX-Seq strategy. A, Oligonucleotides and PCR primers. The sequences underlined with arrows represented the annealing sites of primers. The bases at the 5′ end of nested primers represented the split barcodes. Biotin-labeled primers were used to prepare the biotin-labeled dsDNAs for NIRF-EMSA. B, EMSA-based SELEX-Seq procedure. The oligonucleotides in red, green and blue represented the positive, negative and random dsDNAs, respectively. PCR1, PCR2 and PCR3 were used to amplify the positive, negative and random dsDNAs with primers P-F/R, N-F/R and S-F1-4/R1-4, respectively. a, annealing; e, elongation; b, binding; p, purification; E, EMSA; P, PCR; R0 to R4, Round 0 to Round 4.

To realize SELEX-Seq with the improved strategy, we designed and synthesized three kinds of 64-bp oligonucleotides. After Klenow reactions, these single-stranded oligonucleotides were converted into dsDNAs and were used as positive, negative and random dsDNAs, respectively (Figure 1A). The positive dsDNA contained a known NF-κB binding site (GGGACTTTCC). The negative dsDNA contained no NF-κB binding site. The random dsDNA contained 16-bp random sequences for SELEX selection. At the ends of these dsDNAs, specific primer annealing sequences were designed for their PCR amplification after each round of SELEX selection. The PCR amplifications of positive and negative dsDNAs were used to monitor the specificity of SELEX selection, while the PCR amplification of random dsDNA was used to monitor the selection of random dsDNA and prepare the input DNA for the next round of SELEX selection and the final sequencing (Figure 1B). The positive and negative dsDNA were added into the random dsDNA at the ratio of 1∶10000 before each round of SELEX selection. To avoid amplification of contaminants and code the DNA from each round of SELEX selection, five sets of nested primers were designed to amplify the selected dsDNA after each round of SELEX selection (Figure 1A). EMSA was employed to isolate the protein-bound dsDNA in each round of SELEX selection (Figure 1B). EMSA was also used to detect the enrichment of dsDNAs in the SELEX selections (Figure 1B).

### Preparation and Evaluation of dsDNA used in SELEX-Seq Experiment

**Figure 2:**
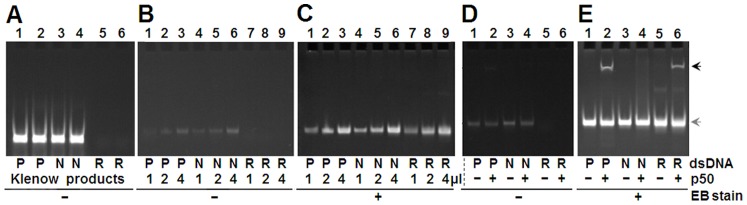
Preparation and evaluation of dsDNAs used in SELEX-Seq. A, Detection of dsDNA products of Klenow reaction with PAGE. B and C, Detection of purified dsDNA with PAGE before and after EB staining. The loading amount of dsDNA: 1, 4, 7: 1 µl; 2, 5, 8: 2 µl; 3, 6, 9: 4 µl. D and E, Detection of dsDNA with EMSA PAGE before and after EB staining. P, positive dsDNA; N, negative dsDNA; R, random dsDNA. Black arrowhead, shifted dsDNA (protein-bound dsDNA); Gray arrowhead, free dsDNA. All PAGE gels were visualized with UV transilluminator.

The dsDNAs used in this study were prepared by converting the chemically synthesized single-stranded oligonucleotides into double-stranded oligonucleotides by using Klenow reaction. The fluorescently labeled positive and negative dsDNAs were prepared by using FAM-labeled primers (P-R and N-R) in Klenow reaction. The prepared dsDNAs were checked with polyacrylamide gel electrophoresis (PAGE) and ethidium bromide (EB) staining (Figure 2A to C). The FAM-labeled positive dsDNA was used to form a control binding reaction for tracking DNA/protein complex in polyacrylamide gel in an EB-free manner. The interactions of these dsDNAs with NF-κB p50 protein were detected with EMSA. It was demonstrated that a shifted band produced by the FAM-labeled positive dsDNA bound by NF-κB p50 protein could be directly visualized on UV transilluminator after EMSA PAGE (Figure 2D). However, after EB staining, a shifted band produced by the random dsDNA bound by p50 protein appeared in gel on UV transilluminator, and the shifted band produced by the FAM-labeled positive dsDNA bound by NF-κB p50 protein became more clear (Figure 2E). These results verified the specificity of positive and negative dsDNAs and the tracking function of FAM-labeled positive dsDNA.

### SELEX Selection and PCR Detection

**Figure 3:**
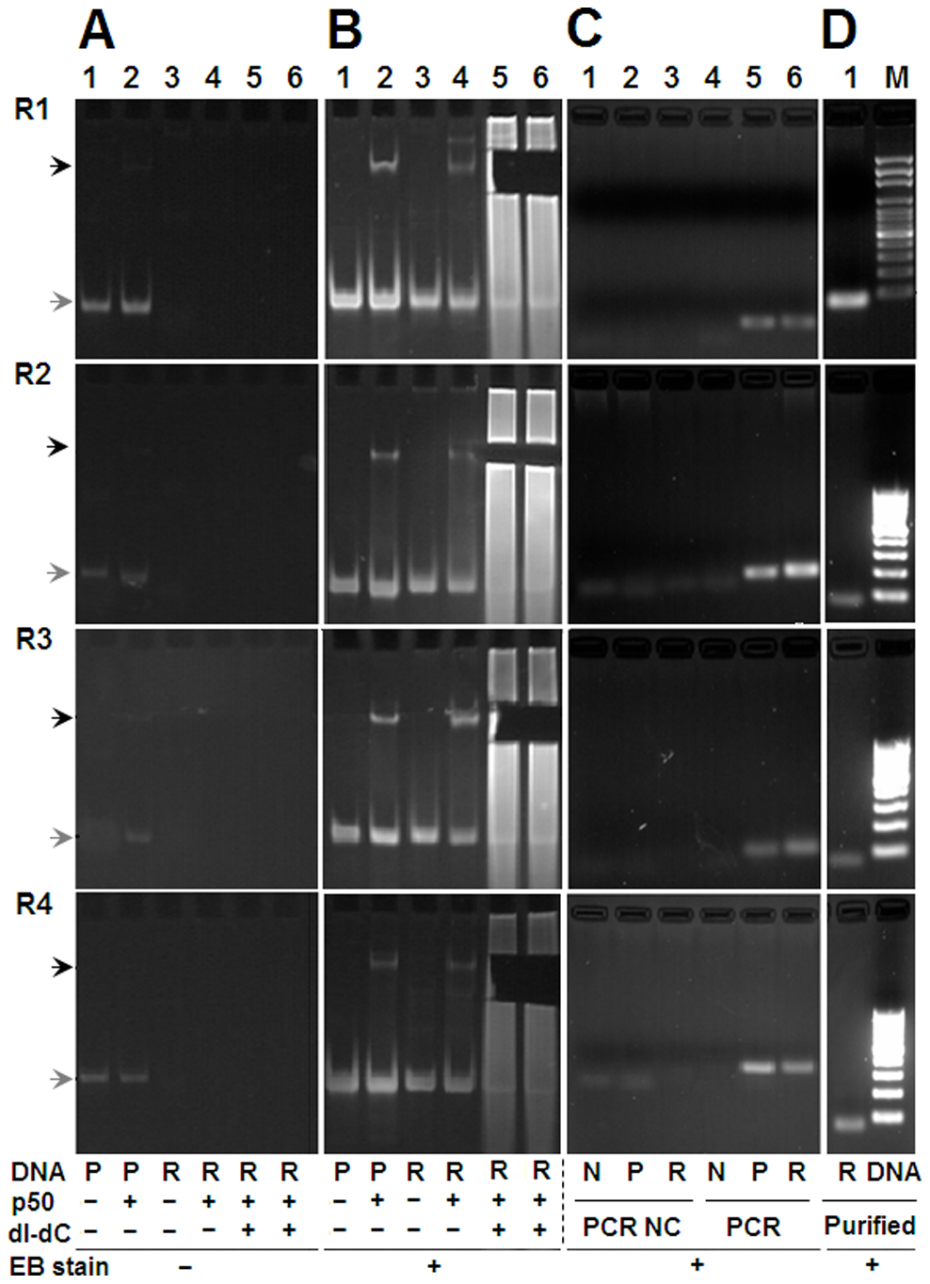
Four rounds of EMSA-based SELEX selection and specificity detection. A and B, Detection of EMSA PAGE gels before and after EB staining. The gel slices containing shifted random dsDNA were cut out before EB staining. Components of EMSA reactions were shown under the images. P, positive dsDNA; R, random dsDNA. Black arrowhead, shifted dsDNA; Gray arrowhead, free dsDNA. C, PCR detection of the SELEX-selected dsDNA. NC, PCR negative controls; N, negative dsDNAs. The selected random dsDNAs were amplified with the nested primers in various SELEX rounds (S-F1/R1 in R1, S-F2/R2 in R2, S-F3/R3 in R3, and S-F4/R4 in R4). R1 to R4, Round 1 to Round 4. D, Detection of the purified PCR products of random dsDNAs. M, DNA maker, the length of the smallest band is 100-bp. All PAGE gels were visualized with UV transilluminator.

Four rounds of SELEX selections were performed. In each round, six gel shift reactions were set up as described in Materials and Methods. The reaction of positive dsDNA plus p50 protein was used as control binding reaction for tracking protein-bound random dsDNAs in gels. Three reactions of random dsDNA plus p50 protein were performed, in which a reaction without polydI-dC was used to confirm that the gel was correctly cut for recovering the protein-bound random dsDNA, and two reactions with polydI-dC were used to recover the protein-bound random dsDNA. To evaluate the specificity of SELEX selections, trace amounts of positive and negative dsDNAs were added into these two reactions. After each round of SELEX selection, the selected random dsDNA was detected with three parallel PCR reactions amplifying positive, negative and random dsDNAs, respectively. The results revealed that the position of the protein-bound random dsDNA was correctly indicated by the control binding reaction and confirmed by the EB staining after gel cutting (Figure 3A and B). The PCR amplifications of positive and negative dsDNAs verified the specificity of SELEX selections (Figure 3C). The PCR amplification of the selected random dsDNAs revealed that the p50-bound random dsDNAs were successfully obtained in each round (Figure 3C). The purified PCR products of the selected random dsDNAs also provided the input DNAs for the next round of selection, final EMSA evaluation and sequencing (Figure 3D).

### Evaluation of SELEX Products with NIRF-EMSA

**Figure 4:**
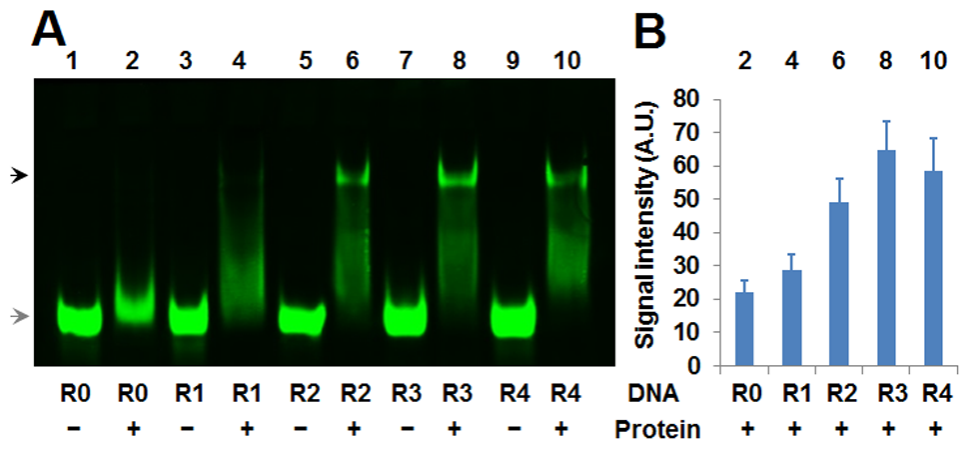
Evaluation of SELEX products with EMSA. A, NIRF-EMSA detection of the selected random dsDNAs from each round. B, Signal quantification of shifted bands of Image A. Components of EMSA reactions were shown under the image. Black arrowhead, shifted DNA; Gray arrowhead, free DNA. R0 to R4, DNAs obtained from Round 0 to Round 4.

The enrichment of random dsDNAs along with SELEX selections were detected with a near infrared fluorescence EMSA (NIRF-EMSA). For this purpose, the selected dsDNAs from each round and the unselected dsDNA were firstly labeled by using PCR amplification with a pair of biotin-labeled universal primers (U-F and U-R, Figure 1). The purified PCR products were used as EMSA probes. The results of NIRF-EMSA demonstrated that the random dsDNAs were gradually enriched with SELEX selections (Figure 4). However, after three selection rounds, the signal intensity of the shifted bands no longer increased (Figure 4).

### DNA Sequencing and Data Analysis

To prepare the DNA sample for high-throughput DNA sequencing by using Illumina Solexa Hiseq2000, the purified PCR products from each selection round were pooled in equal molar to form a multiplexed sample. After a two-directional Hiseq2000 sequencing, a total of 10881840 pairs of full-length 64-bp sequences were obtained. The raw data of Illumina Solexa sequencing obtained in this study have been deposited into the Gene Expression Omnibus (GEO) database under accession number [GSE:48660]. After quality control as described in Materials and Methods, 7347151 clean reads were obtained. These reads were sorted according to their barcode. 444108, 1749128, 2333602, 1779507 and 1040806 reads were obtained for Round 0 to 4, respectively. These reads were used to perform *de novo* motif analysis by using MEME.

**Figure 5:**
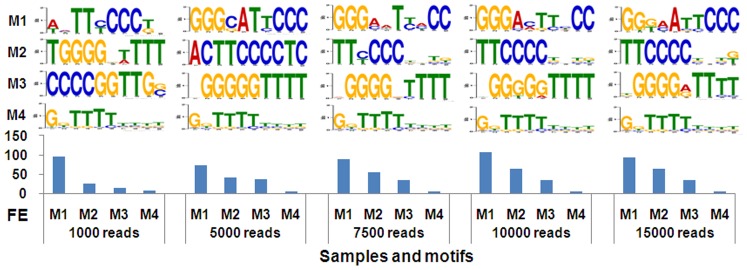
Finding motifs with reads from Round 4 by using MEME. Five motifs were extracted for each of five samples containing various numbers of non-overlapping 16-mer reads from Round 4. The top four motifs were displayed above the histogram of their fold enrichments. M1 to M4, Motif 1 to motif 4. FE, fold enrichment. The title of Y-axis of motif logos was “bits” and the labels were 0, 1 and 2. The labels under the motif logos were “1” to “10” from left to right that referred to the positions of nucleotides in a motif.

Due to large reads data, motif analysis was performed with small amount of reads that were randomly sampled from reads of each round. To determine the optimal sample size, five samples containing non-overlapping 1000, 5000, 7500, 10000 and 15000 reads from Round 4 were firstly used to perform *de novo* motif analysis. Because most previous studies identified NF-κB binding sites as 10-bp sequence [40], five motifs at the length of 10 bp were extracted. The results were shown in Figure 5. The fold enrichment of each motif was also calculated as describe in Materials and Methods. It was found that the motifs and their fold enrichments of the samples containing 7500, 10000 and 15000 reads were similar. The most enriched motifs of these samples were similar to the known κB motif (Table S2 in File S1). Therefore, the samples with 10000 reads were used to perform subsequent *de novo* motif analysis.

**Figure 6:**
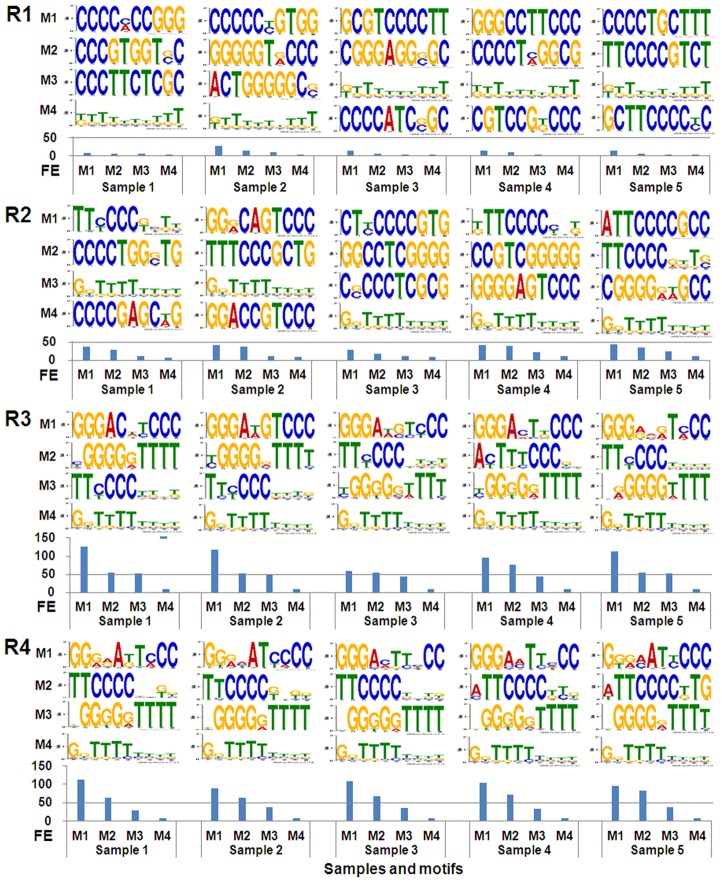
Finding motifs with reads from various rounds by using MEME. Five motifs were extracted for each of five samples containing 10000 non-overlapping reads from Round 1 to 4. The top four motifs were displayed above the histogram of their fold enrichments. M1 to M4, Motif 1 to motif 4. FE, fold enrichment. R1 to R4, Round 1 to Round 4. The title and labels of coordinate axes of motif logos were same as Figure 5.

To check the enrichment of SELEX selection, new *de novo* 10-mer motif analysis was performed with 25 samples of 10000 reads (5 samples for reads of each round). For each sample, five motifs were extracted. The results were shown in Figure 6. The fold enrichment was also calculated for each motif obtained. As a result, it was found that the similar motifs could be obtained for all samples of Round 3 and 4 (Figure 6). In addition, the most enriched motifs for all samples of these two rounds were very similar to the known NF-κB motifs (Table S2 in File S1). Moreover, the sequences with poly(T-C) and poly(G-T) were also significantly enriched in Round 3 and 4. However, no motifs of these kinds were obtained with samples of Round 1 and 2. Nevertheless, the motifs with polyC and polyG were enriched in the first two rounds. This was in agreement with the fact that the NF-κB DNA-binding motifs generally contained polyG at 5′ end and polyC at 3′ end. To further check the enrichment of SELEX selection, the fold enrichments of three motifs obtained with reads samples of Round 3 and 4 (Table S3 in File S1) in Round 1 to 4 were calculated. The results revealed that the Motif 1 was significantly enriched along with the SELEX selection, especially in Round 2 (Table S4 in File S1). However, the remained two motifs were not significantly enriched in the SELEX selections (Table S4 in File S1). Therefore, these two motifs were regarded as the less-specific binders of NF-κB and not used to make the final motif of this transcription factor.

**Figure 7:**
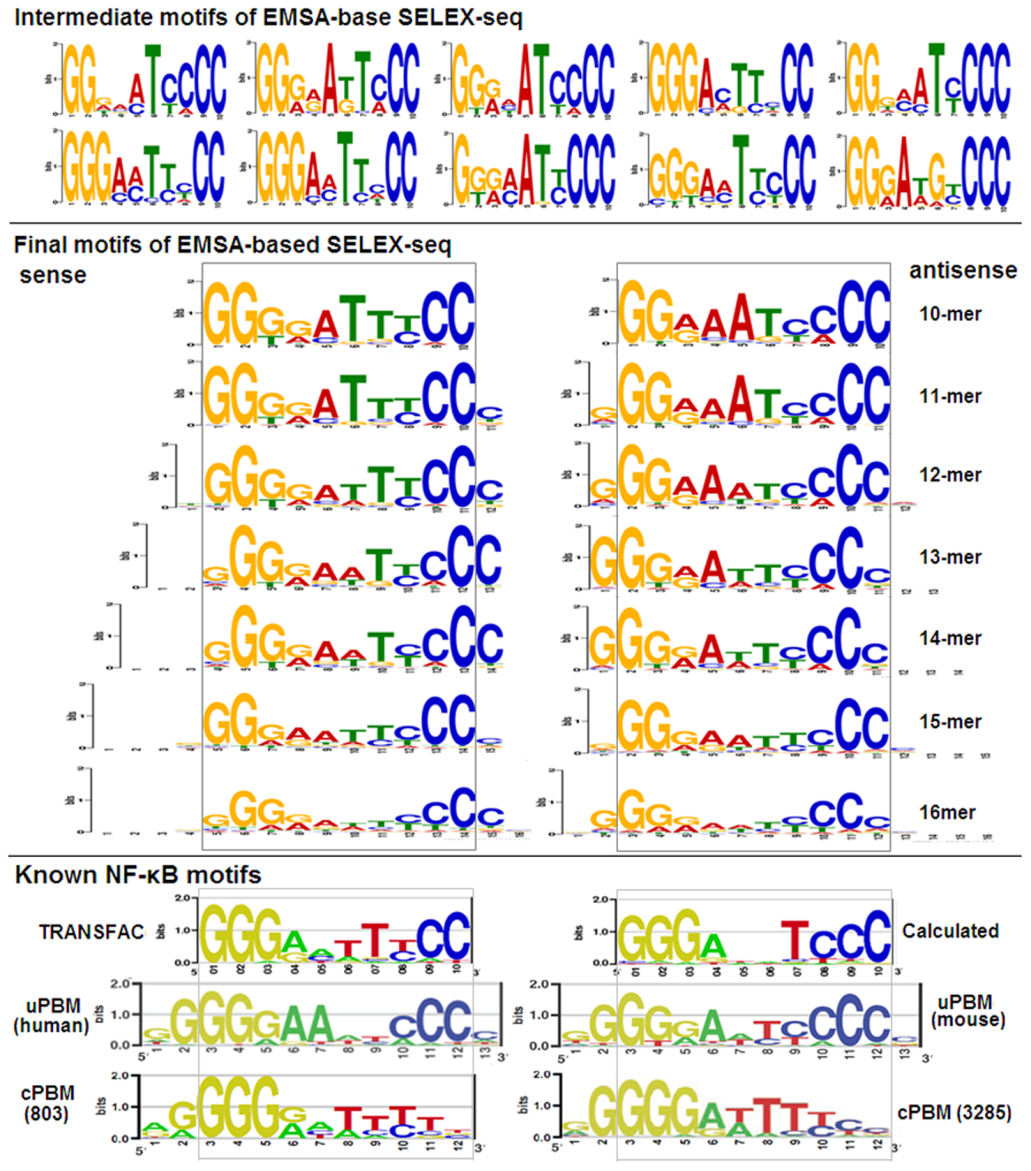
Intermediate and final motifs of NF-κB p50. The intermediate motifs were the most enriched motifs obtained with 10 samples containing 10000 non-overlapping reads from Round 4. The final motifs were obtained with the reads containing 10 intermediate motifs. The known NF-κB motifs came from TRANSFAC, uPBM, custom PBM (cPBM). The title and labels of coordinate axes of motif logos were same as Figure 5.

To obtain the final motif of NF-κB p50 in this study, new *de novo* 10-mer motif analysis was performed with 10 samples containing 10000 non-overlapping reads from Round 4. For each sample, ten motifs were extracted. The most enriched motif of each sample was determined according to fold enrichment. As a result, ten motifs with the highest fold enrichment were obtained; these motifs were regarded as intermediate motifs (Figure 7). Subsequently, all reads containing these intermediate motifs were collected from the reads of Round 4. As a result, 7044 reads were obtained, which contained 6227 different 16-mer sequences. The final motifs were then extracted with these reads. To find the possible variants of motifs, five motifs at the length of 10- to 16-mer were extracted. As a result, only one motif was obtained in finding motifs at various lengths (Figure 7). Furthermore, when extracting motifs at the length 11- to 16-bp, only 11-mer motifs were obtained (Figure 7). In comparison with the 10-mer motif, all 11-mer motifs contained an additional cytosine at their 3′ end. With this cytosine, the 11-mer motifs contained two symmetrically opposed 5-bp half-sites [5′-GGG(G/A)A-3′]. This was in agreement with the motifs of p50 homodimer obtained with universal PBM (uPBM) [41] (Figure 7) and SELEX-cloning sequencing [40]. The final 10-mer motif also resembled the NF-κB motif constructed by TRANSFAC.

In above motif analysis, it was found that all 11-mer motifs contained an additional cytosine at 3′ end in comparison with the 10-mer motif, which suggested that this flanking cytosine contributed to the NF-κB binding to its DNA sites. To verify this speculation, all 16-mer sequences containing a wide-type DNA-binding site of NF-κB, GGGACTTTCC, were collected from reads of Round 4 and classified according to the nucleotides after the site. As a result, it was found that the sequences with cytosine were significantly more than those with three other nucleotides (Figure S1 in File S1). To further verify the speculation, a new EMSA was performed to check the relative binding affinities of NF-κB p50 to two DNA sequences, GGGACTTTCCc and GGGACTTTCCt (Figure S1 in File S1). The result revealed that the protein showed higher binding affinity to the former than the latter (Figure S1 in File S1). These data demonstrated that the cytosine at the 3′ end of 11-mer motifs contributed to the preferential binding of NF-κB to the DNA binding sites with this nucleotide.

### Evaluation of SELEX-Seq Data with Known NF-κB Motifs and Relative Affinity

**Figure 8:**
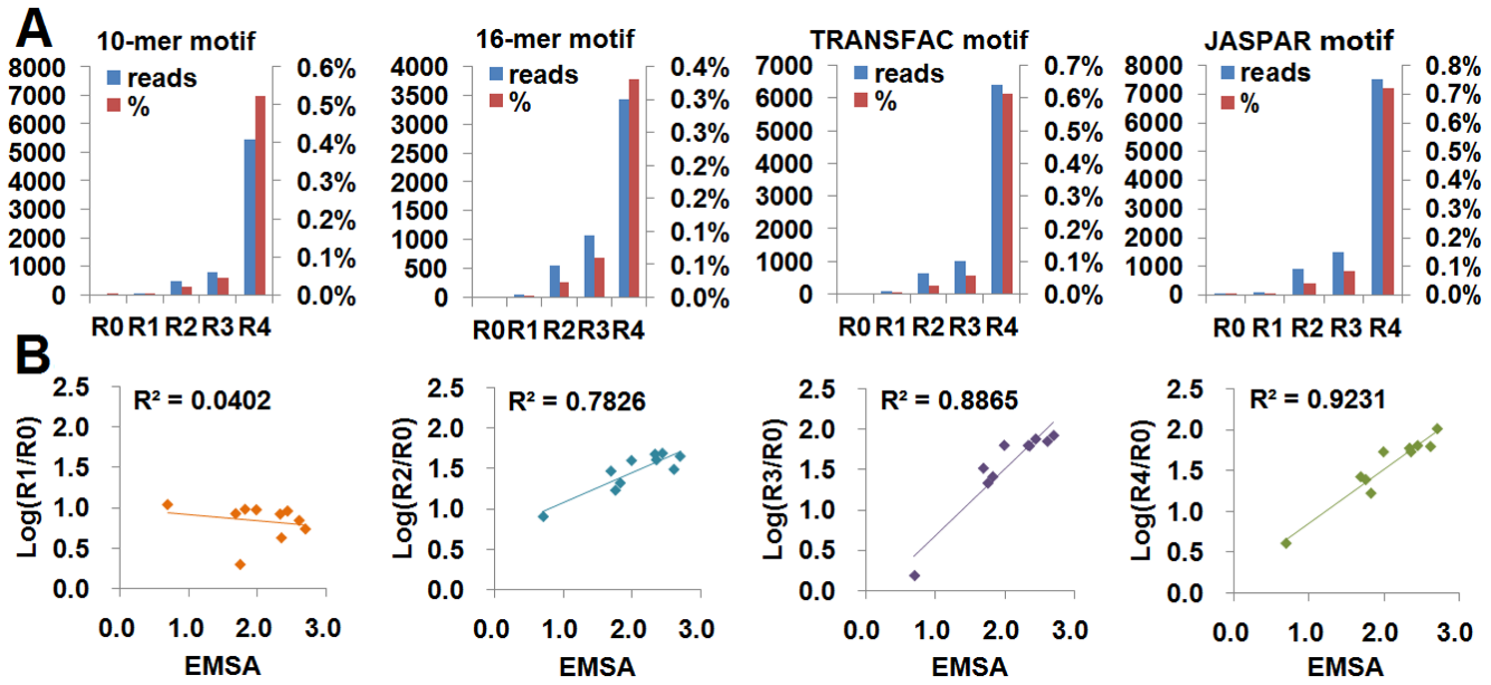
Evaluation of SELEX-Seq with known NF-κB motifs and EMSA-detected affinity. A, Enrichment of NF-κB motifs were shown by the absolute number and the percent of reads that contained a particular motif in a round. The known NF-κB motifs came from TRANSFAC and JASPAR. B, Correlation of the fold enrichment of 10 sequences (10-mer) in four rounds with the relative affinity determined by EMSA. Because the EMSA value was less than 3.0, to compare with EMSA values, the logarithmic value of fold enrichment was calculated.

To evaluate the SELEX-Seq data, the reads contained the known κB motifs (from TRANSFAC entry V$NFKAPPAB_01 and reference [41]; JASPAR entry MA0105) and the final motifs obtained in this study were counted. It was found that the reads containing these motifs gradually increased along with the rounds of SELEX selection (Figure 8A). The κB site GGGACTTTCC was identified in the immunoglobulin (Ig) κ chain enhancer when the transcription factor NF-κB was found for the first time [42]. The enrichment of this wild-type κB site in SELEX-Seq data was also checked. As a result, it was found that this site was enriched 6.83, 39.17, 65.33 and 71.50 fold in Round 1 to 4, respectively.

To further evaluate the SELEX-Seq data, the relative fold enrichment of SELEX-Seq was compared with the relative binding affinities determined by EMSA. Udalova *et al.* examined the binding affinity of NF-κB p50 homodimers to some 10-mer DNA sequences with EMSA [43], it was found that 10 sequences appeared in SELEX-Seq data of all rounds (Table S5 in File S1). The correlation coefficients (R^2^) between the EMSA values and the SELEX-Seq values of four rounds were calculated. The results revealed that the R^2^ value increased with the round of SELEX selection, and an excellent correlation was reached at Round 4 (*R* ^2^>0.9) (Figure 8B). These data demonstrated that the corrected number of a sequence in Round 4 could reflect the relative binding affinities of NF-κB p50 homodimer to the sequence.

## Discussion

In this study, we introduced an improved SELEX-Seq strategy. The strategy was mainly characterized with the following technical improvements. Firstly, this strategy designed a FAM-labeled probe to track the protein-DNA complex in polyacrylamide gels in SELEX selection. Secondly, this strategy introduced a positive and negative sequence into random dsDNAs. Thirdly, this strategy used the nested PCR to amplify the selected DNAs after each round. Fourthly, this strategy designed split barcode to code dsDNAs from various rounds. These technical improvements made this strategy with several advantages over the current SELEX-Seq procedures.

Firstly, the FAM-labeled positive dsDNA facilitated the recovery of protein-bound dsDNAs from polyacrylamide gel without staining gels with EB and other DNA or protein dyes [35]. This improvement prevented dsDNA from dye contamination that may interfere protein-DNA interaction [44], [45]. Moreover, the improvement avoided using radioactively labeled positive dsDNA or even random dsDNA [34], [40] for locating the protein-DNA complex in gels by autoradiography that is dangerous and time-consuming (over 2 hs exposure). Moreover, the end ^32^P-labled dsDNA cannot be directly used to prepare sequencing library by linking sequencing adaptor [40]. Secondly, the specificity and the enrichment of SELEX selection were monitored by PCR amplification of positive and negative sequences added into the selected dsDNAs. The positive dsDNA contained a high-affinity wild-type κB site (5′-aaaGGGACTTTCCaaa-3′), but the negative dsDNA contained no κB site (5′-atagtcgcatgctagc-3′). Therefore, after each round of SELEX selection, the positive dsDNA should be enriched and amplified, but the negative dsDNA should not be enriched and amplified. Thirdly, the specificity of PCR amplification of the selected dsDNAs in each selection round was maintained by using nested PCR. In this study, five pairs of nested PCR primers were designed for amplifying the selected dsDNAs of variant rounds. The specificity of PCR amplification was further protected by low-cycle amplification (20 cycles) with high fidelity Taq polymerase. The nested PCR amplification was also employed by a recent SELEX-Seq study [33]. However, this study combined nested PCR amplification with split barcode technique.

Finally, the split barcode used in this study greatly simplified the SELEX selection. At present, the reported coding approaches used several consecutive nucleotides (such as three- or four- consecutive nucleotides [32], [33]) as barcode (consecutive barcode). However, this study used a few nucleotides added at the 5′ ends of nested primers as barcode. Because the barcode nucleotides were split and set at 5′ ends of a pair of PCR primers, it was named as split barcode. To increase the specificity, each barcode differed from others by at least two nucleotides. In comparison with consecutive barcode, split barcode can greatly simplify the procedures of the SELEX selection. For example, if a four-round SELEX selection is performed with consecutive barcode, five separate random libraries (R0) with different barcodes will have to be prepared for various rounds of SELEX selections and as many as ten selections will have to be performed, because any later rounds (Round 2 to 4) have to be started from a new Round 0. In this case, different later selections are in fact performed with different starting materials (separate random libraries), and the final multiplexed samples are in fact composed of dsDNAs of later selections from different series of SELEX selections. The potential different nonrandomness of various random libraries may affect the calculation of accurate fold enrichment of sequences. However, if the four-round SELEX selection is performed with split barcode, only one random library and four selections will be needed, because the next round can be directly performed with the selected dsDNA from the last round. Moreover, in this case, the starting materials (R0) for all rounds are same which allows calculating the accurate fold enrichment of sequences. In this study, split barcodes of different length were used; however, it can be replaced by split barcodes of the same length, which is still compatible with the nested PCR primers. This strategy allows for increased multiplexing and more flexibility in multiplexing samples. This is particularly important for keeping costs to a minimum when first establishing SELEX-Seq in a laboratory. In addition, this strategy also bypasses the need to sequence a separate ‘Round 0’ for each barcode.

By using the improved SELEX-Seq strategy, this study successful characterized the DNA-binding specificity of a well-known transcription factor, NF-κB. The data analysis revealed that the resulted DNA-binding motifs were identical to the known κB motifs obtained by other studies [35], [40], [46], [47]. The known κB motifs were gradually enriched in the improved SELEX-Seq rounds and the corrected numbers of sequences were in agreement with the binding affinities detected by EMSA (Figure 8). These data demonstrated that the improved SELEX-Seq strategy could be used to characterize the DNA-binding specificity of transcription factors. In this study, the transcription factor NF-κB was used as target protein, because many excellent studies on its DNA-binding specificity have been done by other groups, so that the results obtained by the improved SELEX-Seq strategy can be compared with those obtained by other previous studies.

Until now, there are several SELEX studies on NF-κB p50 homodimers. In 1992, Kunsch *et al.* studied the optimal DNA-binding consensus of p50 homodimer with SELEX-cloning sequencing [40]. They selected the p50 binders with an EMSA-based SELEX procedure from 16-mer random dsDNA and found that the optimal binding consensus of this dimer was GGGGATYCCC [40]. However, due to low throughput of cloning sequencing, they did not provide a DNA-binding profile of this dimer. In 2004, Sooter *et al.* selected the optimal binders of p50 homodimer with a p50 protein-coupled microplate from 30-mer random dsDNA [46], they found that the consensus GGGGATYCCC did not present in the selected sequences of Round 0 and 3, but presented in 10% of the sequences of Round 6. They also found that 93% of the sequenced clones contained recognizable NF-κB binding sites. In 2011, Wong *et al.* characterized the DNA-binding preference of p50 homodimer using a custom PBM (cPBM) containing 803 11-mer sequences within a generalized NF-κB consensus RGGRNNHHYYB [35], and constructed a 11-mer motif of this dimer (Figure 7), in which the right half site was not conserved and preferred polyT (Figure 7). In 2012, Siggers *et al.* also characterized the DNA-binding specificity of p50 homodimer with two different PBMs, one was a cPBM containing 3285 sequences that represented the top-scoring potential κB site sequences, and the other was an universal PBM (uPBM) containing all possible 10-mer sequences [47]. As a result, the motif obtained with 3285-sequence cPBM was almost identical to that obtained with 803-sequence cPBM (Figure 7). However, a different motif was obtained with uPBM that had conserved polyG at the left half site and polyC at the right half site (Figure 7). Moreover, almost identical motifs were obtained for human and mouse p50 homodimers with uPBM (Figure 7). In comparison, the 11-mer motif obtained by this study was almost identical to the motifs obtained by uPBM [41]. Therefore, this study firstly characterized the DNA-binding specificity of p50 homodimer with all possible 16-mer sequences by using an improved SELEX-Seq strategy. The DNA-binding specificity characterized by this study should be useful for analyzing the *in vivo* regulatory elements of NF-κB [35], [47] and designing therapeutic decoys targeted to this transcription factor [48], [49].

Until now, as many as 2000 TFs have been found in the human genome [1]–[3], in which only about 700 TFs were identified as DNA-binding TFs [3], [4]. When these proteins were identified as DNA-binding TFs, their a few wild-type DNA-binding sites were found. For example, when NF-κB was firstly identified as a DNA-binding transcription factor, it was found as a protein that could bind the DNA sequence of GGGACTTTCC in kappa chain of human immunoglobulin [42]. At present, the wild-type DNA-binding sites of almost all known DNA-binding TFs can be found in many TF-related databases such as TRANSFAC, JASPAR, TFDB,TRRD, PAZA, MAPPER, etc., these known wild-type DNA-binding sites can be used for designing the positive binding sequence when profiling the high-resolution DNA-binding spectrum of TFs with this new developed SELEX-Seq strategy. However, for those under-studied factors whose binding specificity is unknown, the positive and negative probes cannot be designed, which will limit the application of this strategy. In this case, a protein binding reaction that contained no polydI-dC may be used as an indicator (such as lane 4 in Figure 3B) for selecting their DNA binders by using the similar EMSA-based SELEX selection. Of cause, under the circumstance, the protein binding specificity of each round of SELEX selections would not be monitored by PCR as performed in this study.

In conclusion, this study provided an improved SELEX-Seq strategy for characterizing the DNA-binding specificities of transcription factors. In comparison with the current SELEX-Seq procedures, this SELEX-Seq strategy had several advantages that significantly facilitated and simplified the SELEX-Seq experiment. This improved SELEX-Seq strategy was fully validated by successfully characterizing the DNA-binding specificity and constructing a high-resolution DNA-binding profile of a well-known transcription factor, NF-κB. Therefore, this study provided a new generalizable EMSA-based SELEX-Seq strategy that would be valuable to laboratories attempting to setup their own SELEX-Seq experiments.

## Materials and Methods

### Preparation of dsDNA

All oligonucleotides used in this study were listed in Table S1 in File S1. To prepare dsDNA, single-stranded oligonucleotides were converted into dsDNAs by Klenow reaction. A 50-µl Klenow elongation reaction was set up as follows: 5 µl 100-µM single-stranded oligonucleotide template, 7.5 µl 100-µM reverse primer, 4 µl 2.5-mM dNTP, 5 µl 10×Klenow buffer, and 26.5 µl sterile ddH_2_O. The reaction was denatured in boiled water bath for 5 min and then slowly cooled to room temperature. The reaction was added 2 µl Klenow enzymes (5 U/µl, MBI Fermentas) and kept at 37°C for 20 min. The Klenow enzyme was inactivated at 75°C for 10 min. The products were purified with Microcon YM-30 (Millipore) and eluted in 25 µl ddH_2_O. The purified dsDNA was detected with electrophoresis of 2% agarose gel and quantified with NanoDrop 1000 (Thermo Fisher).

### Evaluation of dsDNA with EMSA

EMSA reactions were set up as follows (10 µl): 2.0 µl 5×DNA-binding buffer (5×DBB) (50 mM Tris-HCl, pH7.5, 250 mM NaCl, 2.5 mM EDTA, 15 mM MgCl_2_, 5% glycerol, 2.5 mg/ml BSA, 0.25% NP-40, 0.05 mM DTT), 0.5 µl p50 protein (115 ng; Promega) (Figure S2 in File S1), 0.5 µl dsDNA (positive, negative or random, 200 ng), 7.0 µl ddH_2_O. The negative control was performed with no p50 protein (0.5 µl ddH_2_O in place of 0.5 µl p50 protein). The binding reactions were incubated at room temperature for 60 min. The reactions were fractionated by electrophoresis with 6% native PAGE. The gel was detected with UV transilluminator (Bio-Rad). The gel was stained with EB for 30 min and detected again with UV transilluminator.

### Selection of Protein-binding dsDNA with EMSA-based SELEX

In each round of SELEX selection, six protein-binding reactions (all 10 µl) were performed simultaneously. Reaction 1 and 2 contained 2.0 µl 5×DBB, 0.5 µl ddH_2_O or p50 protein (115 ng), 0.5 µl diluted positive dsDNA (200 ng), and 7.0 µl ddH_2_O. Reaction 3 and 4 contained 2.0 µl 5×DBB, 0.5 µl ddH_2_O or p50 protein (115 ng), 0.5 µl each of diluted positive and negative dsDNA (0.02 ng), 0.5 µl random dsDNA (200 ng), and 6.0 µl ddH_2_O. Reaction 5 and 6 contained 2.0 µl 5×DBB, 0.5 µl p50 protein, 0.5 µl each of diluted positive and negative dsDNA (0.02 ng), 0.5 µl random dsDNA (200 ng), and 1.0 µl polydI-dC (0.5 mg/ml; Amersham), and 5.0 µl ddH_2_O. The molar ratio of random dsDNA to protein was maintained at 5∶1 in all TF binding experiments. Before preparing protein-binding reaction, the positive and negative dsDNAs were 1/10000 diluted, so that the ratios of the positive and negative dsDNA to the random dsDNA were 1∶10000. In preparing protein-binding reactions, other components was firstly mixed and incubated at room temperature for 30 min, then dsDNA was added and incubated at room temperature for 60 min.

The protein binding reactions were loaded on 6% native polyacrylamide gel and migrated in 0.5×TBE buffer for 1.5 hs. After electrophoresis, the gel was directly visualized on UV transilluminator and the gel slice containing the shifted dsDNA (Reaction 5 and 6) was excised according to the position of the shifted band of Reaction 2. The gel was then stained with EB for 30 min and visualized again on UV transilluminator. The gel slice was socked in 100 µl diffusion buffer [0.5 M NH_4_Ac, 10 mM Mg(Ac)_2_, 1 mM EDTA, pH8.0, 0.1% SDS] overnight at 37°C. The diffusion buffer was purified with Microcon YM-30 and DNA was eluted in 25 µl ddH_2_O.

In each round of SELEX selection, 6 PCR reactions were performed: PCR reactions for amplifying positive dsDNA (using primer P-F and P-R), negative dsDNA (using primer N-F and N-R) and random dsDNA (using nested primers: R-F1 and R-R1 for Round 1, R-F2 and R-R2 for Round 2; R-F3 and R-R3 for Round 3; R-F4 and R-R4 for Round 4), and the corresponding negative controls. PCR reaction (10 µl) consisted of 2 µl 10×Taq buffer, 0.4 µl 2.5 mM dNTP, 0.4 µl 10 µM forward primer, 0.4 µl 10 µM reverse primer, 0.4 µl Taq polymerase (2.5 U/µl PrimerStar® HS, TaKaRa), 1.4 µl template dsDNA (10 ng) or ddH_2_O (negative control), and 6.4 µl ddH_2_O. The PCR reactions were incubated for 3 min at 94°C, and then subjected to 20 cycles of 94°C 1 min, 60°C 1 min and 72°C 2 min, finally incubated for 5 min at 72°C. The PCR products were detected by electrophoresis with 2% agarose gel. The PCR product amplified with nested primers was purified with Microcon YM-30, and the DNA was eluted in 25 µl ddH_2_O. The purified PCR product was detected by electrophoresis with 2% agarose gel and quantified with NanoDrop 1000 (Thermo Fisher). The purified PCR product was kept at 4°C for final sequencing and also used as input DNA for the next round of SELEX selection.

To prepare a PCR-amplified product of unselected random dsDNA, the random dsDNA obtained with Klenow reaction and used as input DNA of Round 1 was amplified with a 10-µl PCR reaction including: 2 µl 10×Taq buffer, 0.4 µl 2.5 mM dNTP, 0.4 µl 10 µM forward primer (R-F0), 0.4 µl 10 µM reverse primer (R-R0), 0.4 µl Taq polymerase (2.5 U/µl PrimerStar® HS, TaKaRa), 1.4 µl random dsDNA (10 ng, purified Klenow reaction product), and 6.4 µl ddH_2_O. The PCR program was as follows: 94°C 3 min, 20 cycles of 95°C 1 min, 60°C 1 min and 72°C 2 min; 72°C 5 min. The PCR product was detected by electrophoresis with 2% agarose gel, and then purified with Microcon YM-30 and dissolved in 25 µl ddH_2_O. The purified DNA was detected by electrophoresis with 2% agarose gel and quantified with NanoDrop 1000 (Thermo Fisher). The purified DNA was used to prepare the final multiplexed sample for sequencing as DNA of Round 0.

### Evaluation of SELEX Products with NIRF-EMSA

The selected DNAs from each round were detected with NIRF-EMSA. The selected dsDNAs were firstly labeled with biotin via PCR amplification with two universal biotin-labeled primers (Table S1 in File S1). The PCR reaction contained: 4 µl 10×Taq buffer, 0.8 µl 2.5 mM dNTP, 0.8 µl biotin-forward primer, 0.8 µl biotin-reverse primer, 0.8 µl Taq polymerase (2.5 U/µl PrimerStar® HS, TaKaRa), 2 µl template DNA (10 ng), 10.8 µl ddH_2_O. The PCR program was as follows: 94°C 3 min, 20 cycles of 95°C 1 min, 60°C 1 min and 72°C 2 min; 72°C 5 min. The PCR products were purified with Microcon YM-30 and eluted in 25 µl ddH_2_O. The purified dsDNA was detected by electrophoresis with 2% agarose gel and quantified with NanoDrop 1000 (Thermo Fisher). The EMSA reaction (10 µl) consisted of 2.0 µl 5×DBB, 0.5 µl p50 protein (115 ng), 1.5 µl biotin-labeled dsDNA (200 ng), and 6.0 µl ddH_2_O. The negative control was performed with no p50 protein (0.5 µl ddH_2_O in place of 0.5 µl p50 protein). The protein-binding reactions were incubated at room temperature for 60 min. The remained procedures of NIRF-EMSA were performed as previously described [50].

### High-throughput DNA Sequencing and Bioinformatics

The final DNA products from each round of SELEX selection were mixed in equimolar amounts (100 ng) to generate a multiplexed sample (500 ng) for sequencing. The multiplexed sample was sequenced with Illumina Solexa Hiseq2000 in two directions. The obtained reads were filtered and sorted with Perl scripts. Filters included: definite base calling (A, C, G and T), valid barcode and constant regions, and unique random sequence at the length of 16 bp. After quality control, the ‘clean’ data of 16-mer reads was obtained and used to perform the following bioinformatics analyses.

The motif analysis was performed with MEME4.5.0 in parameters of –dna –revcomp –w 10 -nmotifs 5. Some logos were generated using the enoLOGOS web tool [51]. The fold enrichment of a motif was calculated as the ratio of the reads containing the motif in a selected round (Round 1, 2, 3 and 4) to the reads containing the same motif in the non-selected round (round 0). Reads were assessed for the presence of a motif using FIMO of MEME software package with a threshold score of *P*-value of 0.001.

## Supporting Information

File S1 **Supporting figures and tables. Figure S1**. Contribution of cytosine after the binding site of GGGACTTTCC to the DNA binding affinity of NF-κB. A, all 16-mer sequences containing the site of GGGACTTTCC were collected from reads of Round 4 and classified according to the nucleotides flanking the end of the site. B, EMSA analysis of the relative binding affinities of NF-κB p50 to two dsDNA probes of agttgagGGGACTTTCC**T**aggc (a) and agttgagGGGACTTTCC**C**aggc (b). C, the quantified signal intensity of the shifted band. The ends of two dsDNA probes were labeled with biotin. The protein-binding reactions and the EMSA protocol were same as described in Materials and Methods. **Figure S2**. NF-κB p50 protein was separated by SDS-PAGE and visualized by silver staining. Molecular weight marker (MW marker) was indicated on the left. **Table S1**. Oligonucleotides designed for SELEX. **Table S2**. NF-κB motifs reported by other studies. **Table S3**. Three motifs obtained with reads of Round 3 and 4. **Table S4**. Enrichments of three motifs obtained with the reads of Round 3 and 4. **Table S5**. Enrichments of 10 sequences (10-mer) in SELEX-Seq and the relative affinities determined by EMSA.
(DOC)
