# Supplementary material for: An Improved SELEX-Seq Strategy for Characterizing DNA-Binding Specificity of Transcription Factor: NF-κB as an Example
Source: PLoS One. 2013 Oct 10;8(10):e76109. doi: 10.1371/journal.pone.0076109 (PMC3794954; doi:10.1371/journal.pone.0076109)
Supplement: File S1 — Supporting figures and tables. Figure S1. Contribution of cytosine after the binding site of GGGACTTTCC to the DNA binding affinity of NF-κB. A, all 16-mer sequences containing the site of GGGACTTTCC were collected from reads of Round 4 and classified according to the nucleotides flanking the end of the site. B, EMSA analysis of the relative binding affinities of NF-κB p50 to two dsDNA probes of agttgagGGGACTTTCCTaggc (a) and agttgagGGGACTTTCCCaggc (b). C, the quantified signal intensity of the shifted band. The ends of two dsDNA probes were labeled with biotin. The protein-binding reactions and the EMSA protocol were same as described in Materials and Methods. Figure S2. NF-κB p50 protein was separated by SDS-PAGE and visualized by silver staining. Molecular weight marker (MW marker) was indicated on the left. Table S1. Oligonucleotides designed for SELEX. Table S2. NF-κB motifs reported by other studies. Table S3. Three motifs obtained with reads of Round 3 and 4. Table S4. Enrichments of three motifs obtained with the reads of Round 3 and 4. Table S5. Enrichments of 10 sequences (10-mer) in SELEX-Seq and the relative affinities determined by EMSA. (DOC) [file pone.0076109.s001.doc]

**Supporting information**

| Table S1. Oligonucleotides designed for SELEX | |
| --- | --- |
| Oligonucleotide | Sequence (5' →3') |
| Positive oligonucleotide | ACTGTCTGAGGAGTGTCGTGTGCTAAAGGGACTTTCCAAAACGACTCAGCACCACTTCACTCAC |
| Forward Primer (P-F) | ACTGTCTGAGGAGTGTCGTGTGCT |
| Reverse Primer (P-R) | FAM-GTGAGTGAAGTGGTGCTGAGTCGT |
| Negative oligonucleotide | CGTTACGACAGCAGAGTAGTGATGATAGTCGCATGCTAGCCTGTAAGACTCACTCATCTCGGCT |
| Forward Primer (N-F) | CGTTACGACAGCAGAGTAGTGATG |
| Reverse Primer (N-R) | FAM-AGCCGAGATGAGTGAGTCTTACAG |
| Random oligonucleotide | CAAGTCCATTGACGATACACAGGTNNNNNNNNNNNNNNNNGCTTAGAGTATGCTGTCATCGTGC |
| Primer R-F0 | CAAGTCCATTGACGATACAC |
| Primer R-R0 | GCACGATGACAGCATACTCT |
| Primer R-F1 | AAAGTCCATTGACGATACACA |
| Primer R-R1 | ACACGATGACAGCATACTCTA |
| Primer R-F2 | AGAGTCCATTGACGATACACAG |
| Primer R-R2 | AGACGATGACAGCATACTCTAA |
| Primer R-F3 | GAAGTCCATTGACGATACACAGG |
| Primer R-R3 | AAACGATGACAGCATACTCTAAG |
| Primer R-F4 | AAAATCCATTGACGATACACAGGT |
| Primer R-R4 | AAAAGATGACAGCATACTCTAAGC |
| Biotin-U-F | Biotin-TCCATTGACGATACACAGGT |
| Biotin-U-R | Biotin-GATGACAGCATACTCTAAGC |

Table S2. NF-κB motifs reported by other studies

| Motif | Protein | Method | Ref. | Description |
| --- | --- | --- | --- | --- |
|  | **p50p50**  (human) | cPBM |  | Binding models generated using the top affinity binders from custom NF-κB PBM that contains 803 11-mer sequences within the generalized NF-κB consensus RGGRNNHHYYB (50 top-scoring 11-mer sequences). |
|  | p65p50  (human) |
|  | p65p50  (human) | EMSA-seq |  | Binding models generated using the top affinity binders from EMSA-Seq [top 50 (up) and 1000 (down) binders of 11-mer]. |
|  | p65p50  (human) |
|  | **p50p50**  (mouse) | cPBM |  | DNA binding site motifs derived from 25 top-scoring B sites from custom NF-κB PBM experiments. The custom NF-κB PBM contains 3285 nonredundant 10-bp sequences that represent the top-scoring set of potential κB site sequences. |
|  | p65p50  (human) |
| 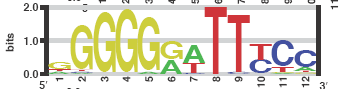 | p65p50  (mouse) |
| 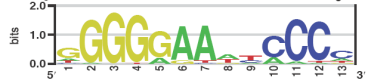 | **p50p50**  (human) | uPBM |  | DNA binding site motifs derived from Universal PBM (uPBM) (containing all 10-bp site) experiments performed on NF-κB dimers. |
|  | **p50p50**  (mouse) |
|  | **p50p50** | Predicted |  | Calculated position weight matrices from protein-DNA complex structures with a novel algorithm. |
|  | p65p50 |
|  | NF-κB | TRANSFAC |  | TRANSFAC entry V$NFKAPPAB_01 |
| 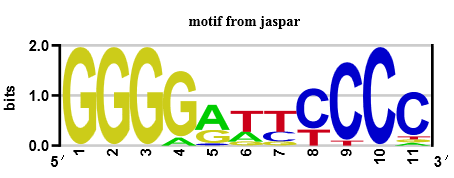 | **p50p50** | JASPAR |  | JASPAR entry MA0105 |

Table S3. Three motifs obtained with the reads of Round 3 and 4.

| Round | sample | Motif 1 | Motif 2/3(Round3);  Motif3 (Round4) | Motif 4 |
| --- | --- | --- | --- | --- |
| 3 | 1 | 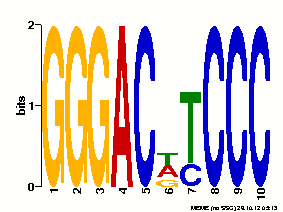  p=5.1e+006; 8 sites | 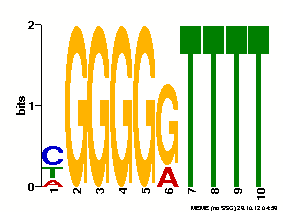  p= 2.5e+006; 10 sites | 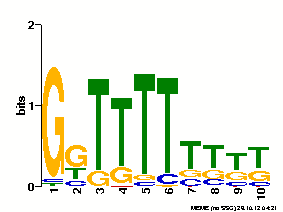  p=1.7e-13424; 9651 sites |
| 3 | 2 | 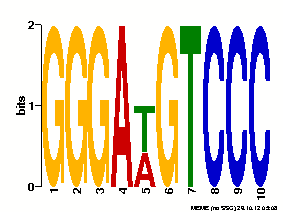  p=2.7e+006; 15 sites | 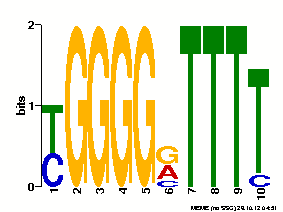  p=6.0e+000; 28 sites | 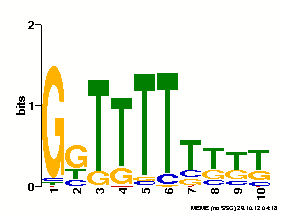  p=9.8e-13455; 9575 sites |
| 3 | 3 | 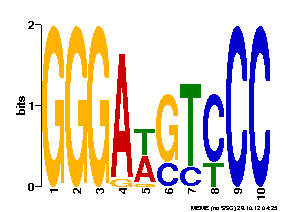  p=2.7e+006; 15 sites | 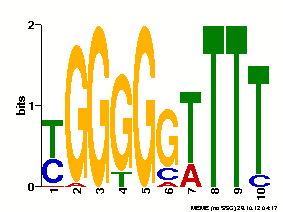  p=6.0e+000; 28 sites | 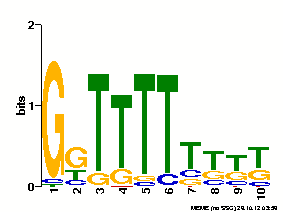  p=9.8e-13455; 9575 sites |
| 3 | 4 | 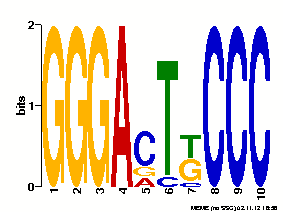  p=7.7e+005; 11 sites | 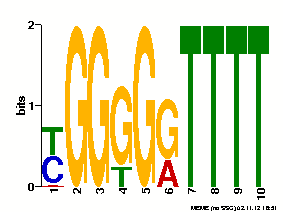  p=3.7e+001; 19 sites | 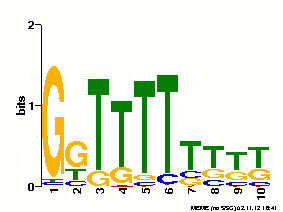  p=8.8e-13540; 9670 sites |
| 3 | 5 | 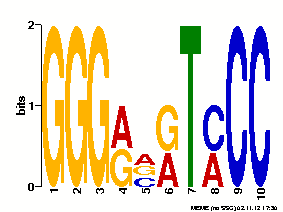  p=2.1e+005; 19 sites | 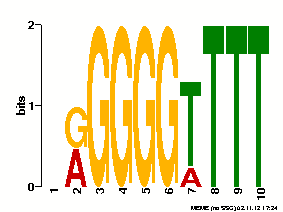  p=1.2e+003; 21 sites | 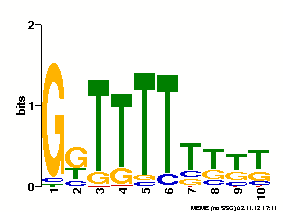  p=4.6e-13229; 9557 sites |
| 4 | 1 | 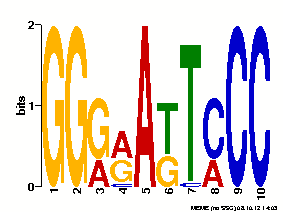  p=8.3e-001; 26 sites | 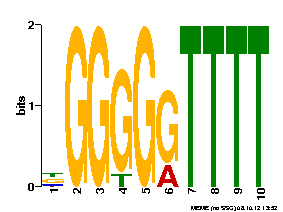  p=8.8e-006; 25 sites | 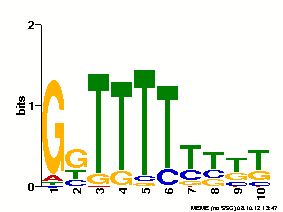  p=1.6e-12927; 9671 sites |
| 4 | 2 | 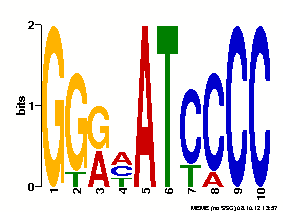  p=2.0e+004; 22 sites | 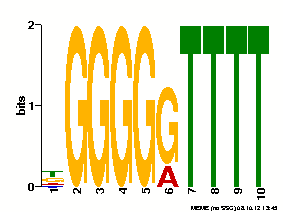  p=9.3e-008; 23 sites | 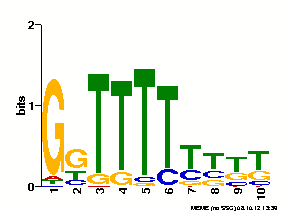  p=3.7e-12919; 9646 sites |
| 4 | 3 | 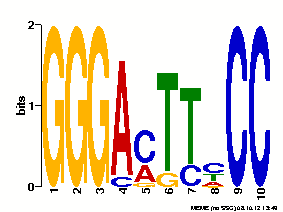  p=4.2e+005; 22 sites | 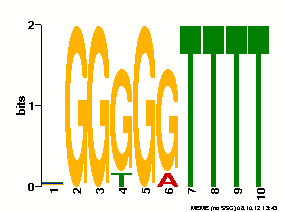  p=4.3e-006; 24 sites | 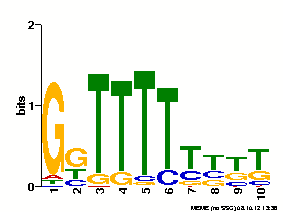  p=3.3e-12894; 9723 sites |
| 4 | 4 | 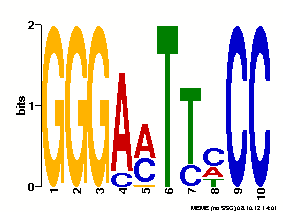  P=2.2e+002; 22 sites | 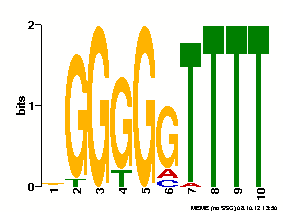  P=1.8e-004; 31 sites | 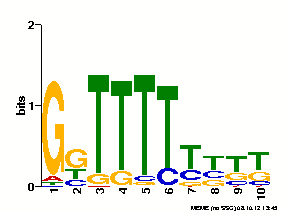  p=3.5e-12804; 9706 sites |
| 4 | 5 | 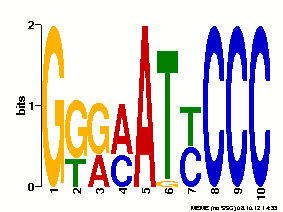  p=5.8e+006; 18 sites | 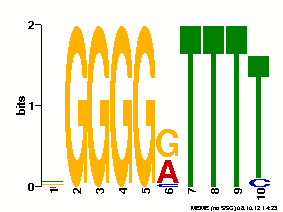  p= 4.4e-007; 29 sites | 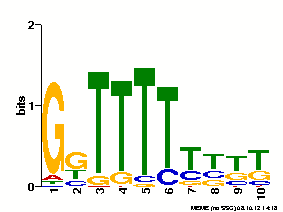  p=1.8e-12926; 9726 sites |

Table S4. Enrichments of three motifs obtained with the reads of Round 3 and 4.

| Motif | Enrichments of motifs in Round 1 to 4 |
| --- | --- |
| Motif 1 in Table S3 | 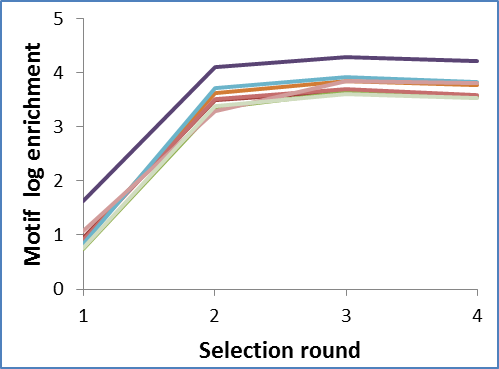 |
| Motif 2/3(Round3) or  Motif3 (Round4) in Table S3 | 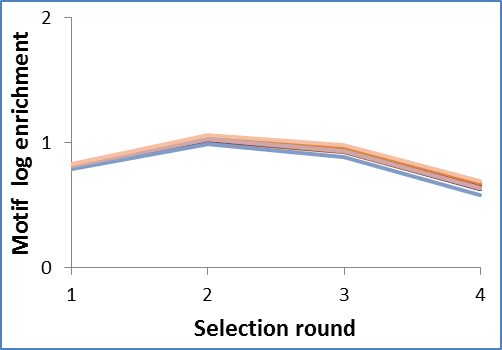 |
| Motif 4 in Table S3 | 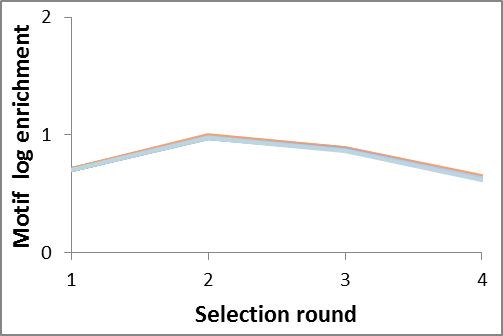 |

**Effect of cytosine after the sequence GGGACTTTCC on the DNA binding affinity of NF-κB**


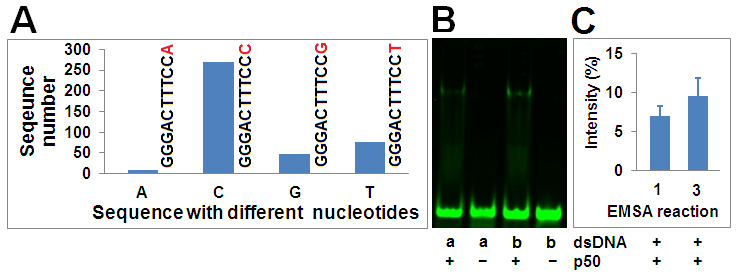


Figure S1. Contribution of cytosine after the binding site of GGGACTTTCC to the DNA binding affinity of NF-κB. A, all 16-mer sequences containing the site of GGGACTTTCC were collected from reads of Round 4 and classified according to the nucleotides flanking the end of the site. B, EMSA analysis of the relative binding affinities of NF-κB p50 to two dsDNA probes of agttgagGGGACTTTCC**T**aggc (a) and agttgagGGGACTTTCC**C**aggc (b). C, the quantified signal intensity of the shifted band. The ends of two dsDNA probes were labeled with biotin. The protein binding reactions and the EMSA protocol were same as described in Materials and Methods.

**Detection of NF-κB p50 protein with SDS-PAGE and silver staining**

The 10% SDS-polyacrylamide gel was prepared and 0.25 μl of NF-κB p50 protein (~60 ng) (Promega) was run (40V for 40 min in stacking gel and 80V for 3h in separating gel) with a molecular weight maker. After electrophoresis, the gel was stained with silver as previously described and imaged with a gel imaging system (Biorad). The result was showed in Figure S2.


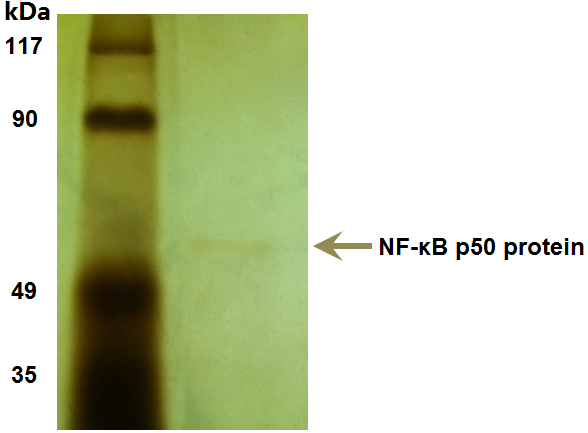


Figure S2. NF-κB p50 protein was separated by SDS-PAGE and visualized by silver staining. Molecular weight marker (MW marker) was indicated on the left.

Table S5. Enrichments of 10 sequences (10-mer) in SELEX-Seq and the relative affinities determined by EMSA

| Sequence | EMSA | SELEX-seq frequency | | | | | SELEX-seq Log value of ratio | | | |
| --- | --- | --- | --- | --- | --- | --- | --- | --- | --- | --- |
|  |  | R0 | R1 | R2 | R3 | R4 | R1/R0 | R2/R0 | R3/R0 | R4/R0 |
| GGGGTGCTCC | 0.699 | 2 | 22 | 16 | 3 | 8 | 1.041 | 0.903 | 0.176 | 0.602 |
| GGGGCTCCCC | 1.690 | 2 | 17 | 59 | 64 | 52 | 0.929 | 1.470 | 1.505 | 1.415 |
| GGGGAGCCCC | 1.756 | 1 | 2 | 17 | 21 | 24 | 0.301 | 1.230 | 1.322 | 1.380 |
| GGGGGCTTCC | 1.826 | 8 | 77 | 168 | 201 | 131 | 0.983 | 1.322 | 1.400 | 1.214 |
| GGGGAGTCCC | 1.991 | 2 | 19 | 80 | 123 | 105 | 0.978 | 1.602 | 1.789 | 1.720 |
| GGGGGATTCC | 2.336 | 5 | 42 | 240 | 307 | 291 | 0.924 | 1.681 | 1.788 | 1.765 |
| GGGGTTCCCC | 2.356 | 11 | 47 | 449 | 666 | 578 | 0.631 | 1.611 | 1.782 | 1.721 |
| GGGGCTTCCC | 2.444 | 6 | 55 | 296 | 442 | 376 | 0.962 | 1.693 | 1.867 | 1.797 |
| GGGGAATCCC | 2.614 | 1 | 7 | 31 | 69 | 61 | 0.845 | 1.491 | 1.839 | 1.785 |
| GGGGATTCCC | 2.704 | 6 | 33 | 271 | 490 | 604 | 0.740 | 1.655 | 1.912 | 2.003 |

The data of relative affinity determined by EMSA came from Reference .

**References**

1. Wong D, Teixeira A, Oikonomopoulos S, Humburg P, Lone IN, et al. (2011) Extensive characterization of NF-kappaB binding uncovers non-canonical motifs and advances the interpretation of genetic functional traits. Genome Biol 12: R70.

2. Siggers T, Chang AB, Teixeira A, Wong D, Williams KJ, et al. (2012) Principles of dimer-specific gene regulation revealed by a comprehensive characterization of NF-kappaB family DNA binding. Nat Immunol 13: 95-102.

3. Alamanova D, Stegmaier P, Kel A (2010) Creating PWMs of transcription factors using 3D structure-based computation of protein-DNA free binding energies. BMC Bioinformatics 11: 225.

4. Gregan J, Riedel CG, Petronczki M, Cipak L, Rumpf C, et al. (2007) Tandem affinity purification of functional TAP-tagged proteins from human cells. Nat Protoc 2: 1145-1151.

5. Udalova IA, Mott R, Field D, Kwiatkowski D (2002) Quantitative prediction of NF-kappa B DNA-protein interactions. Proc Natl Acad Sci U S A 99: 8167-8172.
